# Investigating the effects of wind loading on three dimensional tree models using numerical simulation with implications for urban design

**DOI:** 10.1038/s41598-023-34071-5

**Published:** 2023-05-04

**Authors:** Majid Amani-Beni, Mahdi Tabatabaei Malazi, Kaveh Dehghanian, Laleh Dehghanifarsani

**Affiliations:** 1 School of Architecture, Southwest Jiaotong University, Chengdu, 611756 China; 2 Department of Mechanical Engineering, Faculty of Engineering, Istanbul Aydin University, Istanbul, 34295 Turkey; 3 Department of Civil Engineering, Faculty of Engineering, Istanbul Aydin University, Istanbul, 34295 Turkey; 4 Chengdu, China

**Keywords:** Environmental sciences, Environmental chemistry

## Abstract

In this study, the effects of wind on an Eastern Red Cedar were investigated using numerical simulations. Two different tree models were proposed, each with varying bole lengths and canopy diameters. A total of 18 cases were examined, including different canopy diameters, bole lengths, and wind velocities. Using computational fluid dynamics (CFD) methods, the drag force, deformation, and stress of the tree models were calculated under different wind velocities and geometric parameters. A one-way fluid–structure interaction (FSI) method was used to solve the deformation of the tree. Additionally, velocity and pressure distribution around the tree were obtained. The results indicate that wind velocity and geometric parameters of the tree have a significant impact on deformation, drag force, and stress. As wind velocity increases from 15 to 25 m/s, the force on the tree increases substantially. The results also show that the diameter of the canopy has a bigger effect on stress and strain than the bole length. This study provides insights into tree behavior under wind loading for urban planning and design, informing optimal tree selection and placement for windbreak effectiveness and comfortable environments.

## Introduction

Ocean levels are rising due to climate change, which is creating an unprecedented amount of catastrophic weather occurrences^1,2^. The number of people on earth is still rising^3^, and urban areas are developing and growing^4^. Most of these listed objectives may be met with environmental and natural methods^5^. Prior studies have linked environmental issues with solutions to achieve the UN SDGs, including plant protection^6^, soil and soil science^7^, and the avoidance of land degradation^8^. The literature has shown a connection between trees, green areas, and mortality^9–12^. In one study, the authors linked the infestation and demise of ash trees in counties throughout the United States to an increase in cardiovascular and respiratory fatalities^13^. The correct mature kinds of trees planted in the right places can help reduce the particle problem and other types of air pollution, which can help to lower mortality and morbidity in our urban areas. Moreover, trees have impacts on urban planning, design, landscape architecture, and urban climate. Urban trees have the ability to change the temperature, humidity, wind speed, and contaminants in the air^14^. According to some studies^15–20^, certain specific characteristics, such as the structure and density of the tree, size, shape, and color affect the environment. According to the ways that tree canopies reflect, transmit, and absorb solar radiation and regulate wind speed, they may adapt to microclimates^21^. In tropical climates, the potential for shifting wind patterns and shadows will alter the microclimate and enhance thermal comfort for people^22–24^. Rows of trees or bushes that act as windbreaks can lessen the wind’s power. They have the ability to lower soil erosion, boost agricultural yields, and shield cattle from the heat and cold. Buildings and roads can be protected from drifting snow by windbreaks. They enhance the environment and give wildlife access to the landscape as well as habitat. Windbreaks can be used as food and wood sources. They can transfer of sand particles, microclimate and soil conditions^25^. Windbreaks can be made of natural materials, such as trees and vegetation, or industrial materials, like a concrete wall. Vegetation barriers are used to reduce noise pollution and high wind velocity in cities. Despite the low expense of these shelters^26^, they may not provide enough protection. If the protected building is a low-rise one, one row of trees may be enough to provide enough protection. For higher cases or larger areas, several rows may provide enough protection^27^. Natural vegetation and especially trees mitigate soil erosion. Soil erosion induces loss of soil nutrients and water, water pollution, and global change^28^. Soil degradation is one of the most noteworthy natural issues in the world. In semiarid Mediterranean regions, the dry climate leads to a low level of plant cover, which, in turn, leads to low soil structure development^29^. In China, there are about 3.3 million km^2^ of desertified lands caused by wind erosion^30,31^. In combination with field tests, numerical models may give more noteworthy knowledge about the wind-induced drag acting on trees beneath distinctive scenarios. Later improvements in computational fluid dynamics (CFD), experimental and field studies have looked for the complex and energetic wind–tree interaction, in order to gauge the force that the tree can persevere in given areas and evaluate the tree’s situation and the hazard of tree failure. Since the 1950s, researchers have examined the optimization of vegetative windbreaks and found that efficiency is determined by numerous contributing components. Windbreak height is the major controlling factor, and the length of a windbreak ought to be at least ten times its height^32^. Moreover, windbreaks perpendicular to the approaching speed were found to be more compelling^33^. A windbreak’s width can also impact its viability (number of rows) and is additionally critical for shielding^34^. Last but not least, the geometry and density of trees are key factors^35^. Because of their wide range of agricultural applications, dense canopies have been the focus of many recent transport process studies^36–39^. Trees with heights much greater than the spacing of individual plants characterize dense canopies. Pietri et al.^40^ conducted experiments on dense and sparse canopies to investigate the effect of canopy density on turbulence characteristics within and above canopies. In addition to dense tree canopies, recent research has focused on windbreaks and forest clearings^41,42^. The horizontal spacing between plants in this type of canopy is greater than the plant height. These types of canopies are useful for erosion control and shelter. Many studies have been carried out to better understand windbreaks and their effects on atmospheric surface-layer flow fields. Speckart and Pardyjak^43^ developed and implemented models for mean and fluctuating velocities around a windbreak in a simple, empirically based CFD code. Mayaud et al.^44^ investigated the effects of a single tree, a grass clump, and a shrub on turbulent wind flow and discovered that wind velocity can be reduced by up to 70% in the lee of vegetation. Leenders et al.^45^ investigated wind velocity patterns and wind-induced soil erosion in the vicinity of five different types of vegetation. Their findings revealed that wind velocity was reduced close to the soil surface for shrubs but increased around the trunk for trees. Numerical studies of sparse canopies have used different methods, including Reynolds Averaged Navier–Stokes (RANS) and Large-Eddy Simulation (LES) solvers. Numerous studies have also been done on numerical wind flow predictions. These were regarded as inefficient in the execution of numerical flow computations because complete descriptions of the geometries of twigs and leaves in tree crowns need a significant amount of computer time. To investigate how the urban canopy layer contributes to the development of a nighttime urban boundary layer, Uno et al.^46^ devised a second-order turbulence model. Hiraoka^47^ used ensemble-averaging and spatial averaging approaches based on the eddy-viscosity concept to simulate flows in plants and urban canopies. In order to properly depict the crown penetration characteristics, Shaw and Schumann^48^ suggested adding the proper source terms to the momentum equations used in flow simulations of the spatial sections of tree crowns. The combined effects of the drag coefficient, leaf density, and combined velocities resulted in the newly introduced drag terms. A 2-D numerical model was also used by Wilson and Flesch^49^ to simulate flow fields in forests. Comparatively, the in-field results and forecasted wind velocity profiles were in good agreement. Wei et al.^50^ studied the effect of meteorological parameters in winter and summer and human thermal comfort in different landscapes of an urban park in China. They examined several factors and concluded that in summer, the most comfortable type of landscape space is wood. Wang et al.^51^ showed the correlation between green space and improvements in adult health. In a similar study, Liu et al.^52^ investigated the impact of environmental parameters such as trees on the mental and physical health of residents. Drag force, deformation, and stress of a three-dimensional T-shaped flexible beam were investigated numerically and experimentally by Malazi et al.^53^. They used a two-way fluid–structure interaction (FSI) numerical method for all simulations. A system coupling was employed to connect the fluid and solid domains. Furthermore, an open channel with a high-quality camera was used for the measurement of deformation on a T-shaped flexible beam. Between the numerical and experimental methods, good results were obtained.

In this study, a systematic investigation of windbreaks using CFD simulations has been conducted, and the Eastern Red Cedar was selected as a model tree to evaluate various parameters. The study begins by introducing the numerical simulation methods, followed by a discussion of the analysis results and the selection of optimal design parameters. Finally, conclusions are presented, including a discussion of the novelty and limitations of the study. Additionally, the study provides valuable insights into the behavior of trees under wind loading and the key parameters that influence the performance of trees as windbreaks. This can inform the selection and placement of trees in urban areas to optimize their effectiveness in reducing wind velocities and creating more comfortable environments for residents. Furthermore, the examination of tree deformation and stress on soil can assist in selecting appropriate ground for trees with different bole lengths and crown diameters, which is crucial for selecting soil in parks.

## Materials and methods

### Governing equations and numerical methods

Ansys Workbench-system coupling was applied for the solution of one-way fluid–structure interaction. First, the fluid domain calculates in the Ansys Fluent part, and the solid domain computes in the Ansys Mechanical part. Then a coupling system connects the two parts together. The forces obtained on the fluid side are transferred to the solid side, and then the displacement, stress, and strain of the solid part can be calculated. In this study, the realizable k–ε turbulence model was used for solving the fluid domain, and the static structure method was employed for solving the solid domain. Details of the models were explained below.

### Computational fluid dynamics (CFD)

The turbulent flow simulation in the three-dimensional computational fluid domain was implemented using the realizable k-ɛ turbulence model^53,54^. The continuity and momentum formulas can be shown as:

1 $$\frac{\partial \rho }{\partial t}+\frac{\partial \left(\rho {u}_{i}\right)}{\partial {x}_{i}}=0,$$

2 $$\frac{\partial (\rho {u}_{i})}{\partial t}+\frac{\partial (\rho {u}_{i}{u}_{j})}{\partial {x}_{j}}=-\frac{\partial P}{\partial {x}_{i}}+\rho {g}_{i}+\frac{\partial }{\partial {x}_{j}}\left(\mu +{\mu }_{t}\right)\left(\frac{\partial {u}_{i}}{\partial {x}_{j}}+\frac{\partial {u}_{j}}{\partial {x}_{i}}\right)+{S}_{i},$$

where $\rho$ is the density. ${u}_{i}\, and\, {u}_{j}$ represent the average velocity component of the fluid. P is pressure, *S*_*i*_ is the source term for the momentum equation, $\mu$ represents the dynamic viscosity, ${\mu }_{t}$ represents the eddy viscosity, and it is calculated as ${\mu }_{t}=\rho {C}_{\mu }\frac{{k}^{2}}{\varepsilon }$. The transport equations for $k\, and \,\varepsilon$ for the realizable *k*–*ɛ* model can be written as,

3 $$\frac{\partial }{\partial \mathrm{t}}\left(\mathrm{\rho k}\right)+\frac{\partial }{\partial {\mathrm{x}}_{\mathrm{i}}}\left(\mathrm{\rho k}{\mathrm{u}}_{\mathrm{i}}\right)=\frac{\partial }{\partial {\mathrm{x}}_{\mathrm{j}}}\left[\left(\upmu +\frac{{\upmu }_{\mathrm{t}}}{{\upsigma }_{\mathrm{k}}}\right)\frac{\partial \mathrm{k}}{\partial {\mathrm{x}}_{\mathrm{j}}}\right]+{\mathrm{G}}_{\mathrm{k}}+{\mathrm{G}}_{\mathrm{b}}-\mathrm{\rho \varepsilon }-{\mathrm{Y}}_{\mathrm{M}}+{\mathrm{S}}_{\mathrm{k }},$$

4 $$\frac{\partial }{\partial t}\left(\rho \varepsilon \right)+\frac{\partial }{\partial {x}_{i}}\left(\rho \varepsilon {u}_{i}\right)=\frac{\partial }{\partial {x}_{j}}\left[(\mu +\frac{{\mu }_{t}}{{\sigma }_{\varepsilon }})\frac{\partial \varepsilon }{\partial {x}_{j}}\right]+{C}_{1\varepsilon }\frac{\varepsilon }{k}{(G}_{k}+{{C}_{3\varepsilon }G}_{b})-{C}_{2\varepsilon }\rho \frac{{\varepsilon }^{2}}{k}+{S}_{\varepsilon },$$

where *k* represents the turbulent kinetic energy. *ɛ* represents the rate of dissipation. ${G}_{k}$ is turbulent kinetic energy generation, ${G}_{b}$ is turbulent kinetic energy generation, and ${Y}_{M}$ is fluctuating dilatation contribution to the overall dissipation rate. The model constants for the realizable *k*-*ɛ* turbulence model can be written ${C}_{1\varepsilon }=1.44$, ${C}_{2\varepsilon }=1.92$, ${\sigma }_{k}=1.0$, ${C}_{\mu }=0.09$, and ${\sigma }_{\varepsilon }=1.3.$

### Computational structural dynamics (CSD)

A three-dimensional flexible solid structure's deformation is explained by the equation of motion^53^, which can be given as follows:

5 $$\left[\mathrm{M}\right]\left\{\ddot{u}\right\}+\left[\mathrm{C}\right]\left\{\dot{u}\right\}+\left[\mathrm{K}\right]\left\{u\right\}=\left\{F\right\},$$

where [M] represents the structural mass matrix, [C] represents the structural damping matrix, [K] represents the structural stiffness matrix, and $\left\{F\right\}$ represents the applied load vector acting on the solid structure caused by fluid. $\left\{\ddot{u}\right\}$ represents the nodal acceleration vector, $\left\{\dot{u}\right\}$ represents the nodal velocity vector, and $\left\{u\right\}$ represents the nodal displacement vector.

### Computational model and physical conditions

Eastern Red Cedar has been selected as the tree model. This wood type has very low shrinkage. This species is lightweight, moderately soft, and low in strength when used as a beam or post, and low in shock resistance. The heartwood is very resistant to decay^55^.

**Figure 1:**
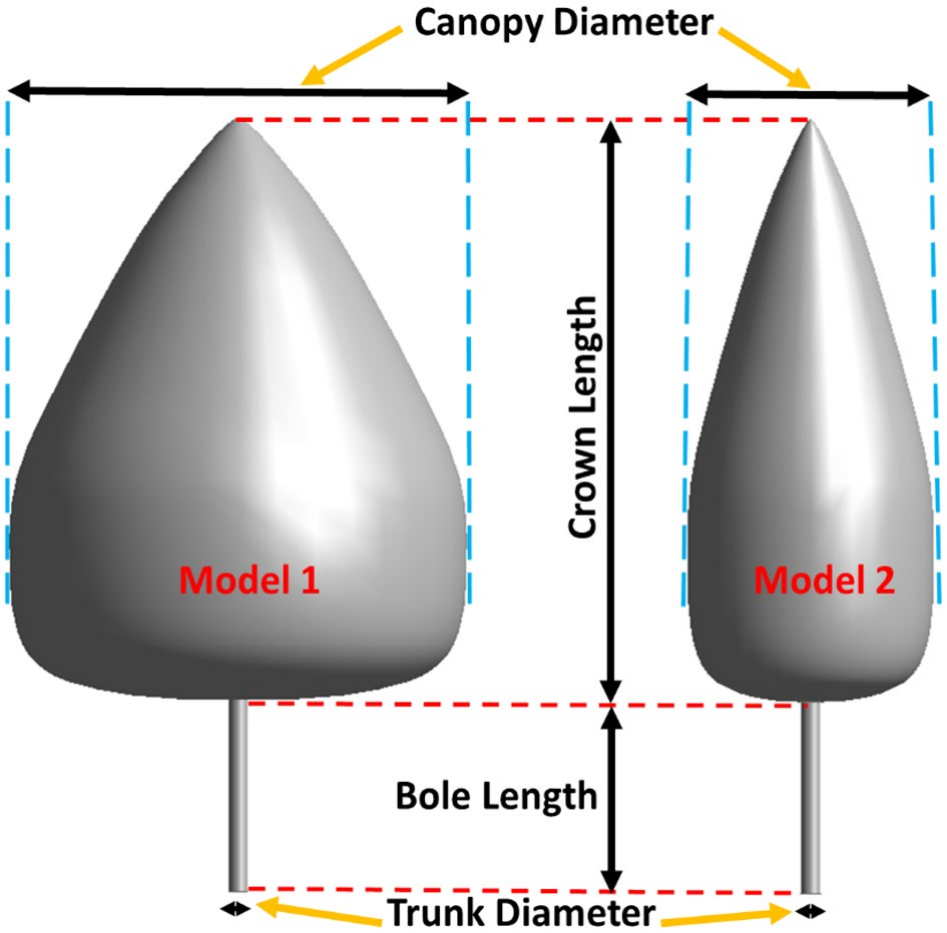
Schematic diagram of the numerical tree models.

The present study investigates two three-dimensional models of an Eastern Red Cedar tree at various geometry parameters numerically (Fig. 1). The canopy diameter of model 1 and model 2 is 2.432 m and 1.216 m. Both models 1 and 2 are pursued at three various bole lengths (0.5 m, 1.0 m, and 1.5 m) and three various wind velocities (15 m/s, 20 m/s, and 25 m/s) with a constant trunk diameter (0.1 m) and a constant crown length (3 m). In this study, 18 different models are analyzed. The details of the numerical simulations carried out in this study are represented in Table 1. The characteristics of air, trees, and soil have been selected as shown in Table 2.

**Table 1:** Numerical simulations run in the present study.

Model	Canopy diameter (m)	Bole length (m)	Trunk diameter (m)	Crown length (m)	Wind velocity (m/s)
1	2.432	0.5	0.1	3	15
1	2.432	1	0.1	3	20
1	2.432	1.5	0.1	3	25
2	1.216	0.5	0.1	3	15
2	1.216	1	0.1	3	20
2	1.216	1.5	0.1	3	25

**Table 2:** Characteristics of wind, tree, and soil in numerical simulations.

Air	
Density $(\rho )$	1.2754 kg/m^3^
Dynamic viscosity (µ)	1.81 × 10^–5^ kg/m^−s^
Tree	
Density (${\rho }_{s})$	336 kg/cm^3^
Young’s modulus (*E*)	4500 MPa
Poisson’s ratio ($\nu )$	0.403
Soil	
Density (${\rho }_{s})$	2170 kg/cm^3^
Young’s modulus (*E*)	43.4 MPa
Poisson’s ratio ($\nu )$	0.354

**Figure 2:**
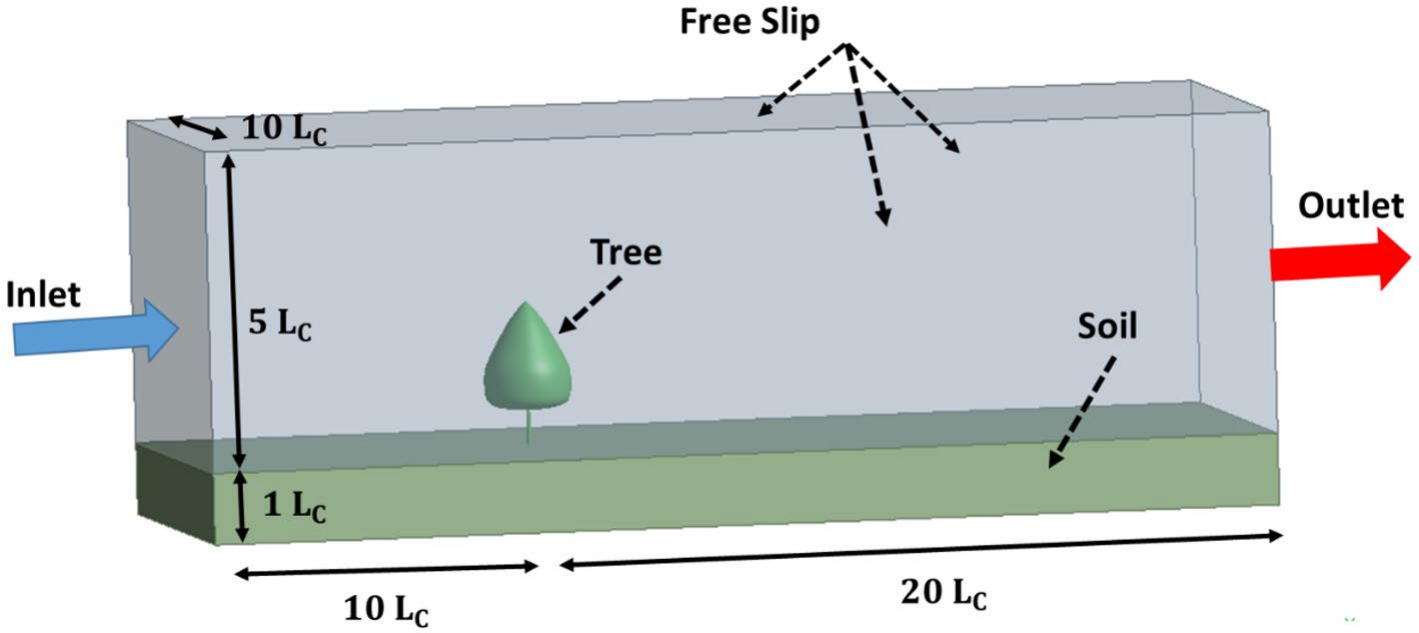
Details regarding the solution’s domain and boundary conditions.

Figure 2 demonstrates the typical model of the computational domain and boundary conditions. A single tree has been modeled in this analysis. L_c_ denotes the crown length of the tree (3 m). The height, length, and width of the domain are defined as 6 L_c_, 30 L_c_, and 10 L_c_. The velocity inlet boundary condition is located at 10 L_c_ upstream of the tree, and the pressure outlet boundary condition is located at 20 L_c_ downstream of the tree. The free slip boundary condition is selected for the upper part of the model.

**Figure 3:**
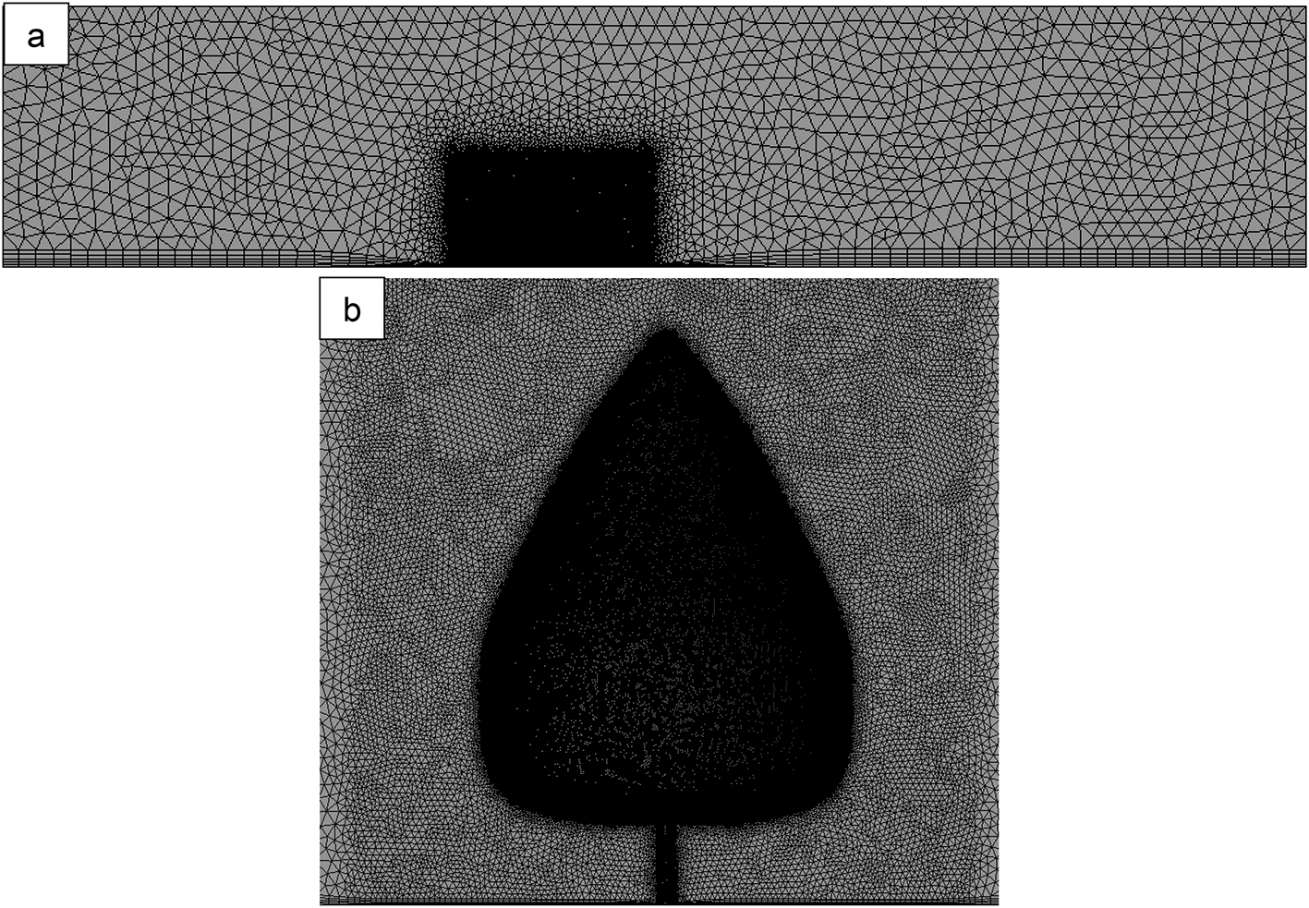
Computational mesh of computational fluid domain (**a**), enlarged view around tree surface (**b**).

Tetrahedron and prism with triangle base mesh were used in the computational fluid domain with high-quality mesh near walls, and tetrahedron mesh is applied in the computational solid domain. As presented in Fig. 3, nearly 13 million elements are used to solve the computational fluid domain, and 6 million elements are applied to solve the solid computational domain.

### Simulation methodology and ethical considerations

This study was based solely on computer simulation and did not involve the use of real Red Cedar plants or any experimentation with plants. This simulation had no conflict with relevant institutional, national, and international guidelines and legislation regarding the ethical use of living organisms in research.

## Results and discussion

### Drag force study

Drag force can act on a body when it is placed in the fluid flow^53^. Drag force on a tree during wind blowing can be calculated using Eq. (6).

6 $${F}_{D}={F}_{D\_pressure}+{F}_{D\_viscous}=\oint P{\widehat{n} .\widehat{e}}_{d}dS+\oint {\tau }_{w}{\widehat{t} .\widehat{e}}_{d}dS,$$

where, ${F}_{D\_pressure}$ is pressure drag, ${F}_{D\_viscous}$ is viscous drag, $p$ is the pressure, and $\tau$ is the wall share stress.

Once the drag force has been computed, the drag coefficient can be calculated using Eq. (7).

7 $${C}_{D}=\frac{{F}_{Drag}}{\frac{1}{2}\rho {U}^{2}A},$$

where* C*_*D*_ is the drag coefficient, ${F}_{Drag}$ is the total drag force, *ρ* is the density of the fluid,* U* is the velocity of the fluid, and *A* is the characteristic area of the body (tree frontal area).

Table 3 represents the comparison of the drag coefficient results between this study and others when the wind velocity is 20 m/s. It was realized that Eastern Red Cedar models 1 and 2 presented very close drag coefficients to real trees.

**Table 3:** Drag coefficient for various species of tree at wind velocity 20 m/s.

Tree species	Drag coefficient
Model 1 (bole length = 0.5 m)	0.2880
Model 1 (bole length = 1.0 m)	0.2891
Model 1 (bole length = 1.5 m)	0.2895
Model 2 (bole length = 0.5 m)	0.3112
Model 2 (bole length = 1.0 m)	0.3163
Model 2 (bole length = 1.5 m)	0.3180
Tree^56^	0.26
Tree^57^	0.3–1.0

**Figure 4:**
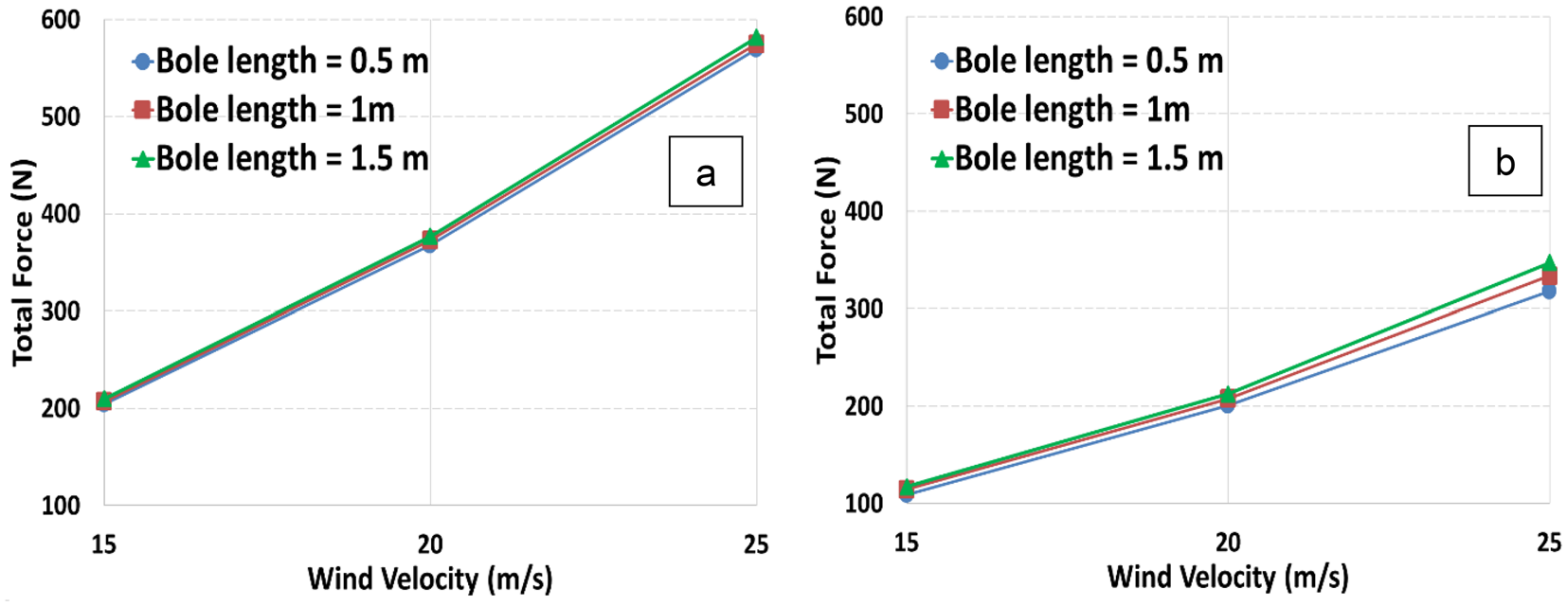
Total drag force varies in relation to various wind velocities for two tree models, model 1 (**a**) and model 2 (**b**).

Figure 4 shows the variation of the total force with respect to the wind velocity in tree models at different bole lengths (0.5 m, 1.0 m, and 1.5 m). Total forces increase with increased wind velocity for models 1 and 2. Total forces are increased by nearly 180% at model 1 when wind velocity changes from 15 to 25 m/s. Moreover, total forces are increased by nearly 200% at model 2 when wind velocity changes from 15 to 25 m/s. The total force has its maximum value when the bole length is 1.5 m for both models. The total force of model 1 is nearly 70% greater than that of model 2 when the wind velocity is 25 m/s. It can be concluded that the canopy diameter has a great influence on the total force. As the diameter increases twice, the total force increases by the same ratio. Moreover, the negligible effect of bole length can be concluded too. A comparison of two models reveals that the canopy diameter has a much more significant effect on the total force results as the canopy diameter decreases.

### Deformation, stress and strain study

Total deformation can be computed in a three-dimensional flexible solid structure numerically. Total deformation is obtained using Eq. (8).

8 $$\mathrm{U}=\sqrt{{\mathrm{U}}_{\mathrm{x}}^{2}+{\mathrm{U}}_{\mathrm{y}}^{2}+{\mathrm{U}}_{\mathrm{z}}^{2}},$$

where ${\mathrm{U}}_{\mathrm{x}}$, ${\mathrm{U}}_{\mathrm{y}}$, and ${\mathrm{U}}_{\mathrm{z}}$ are component deformations in the x, y, and z directions, respectively.

**Figure 5:**
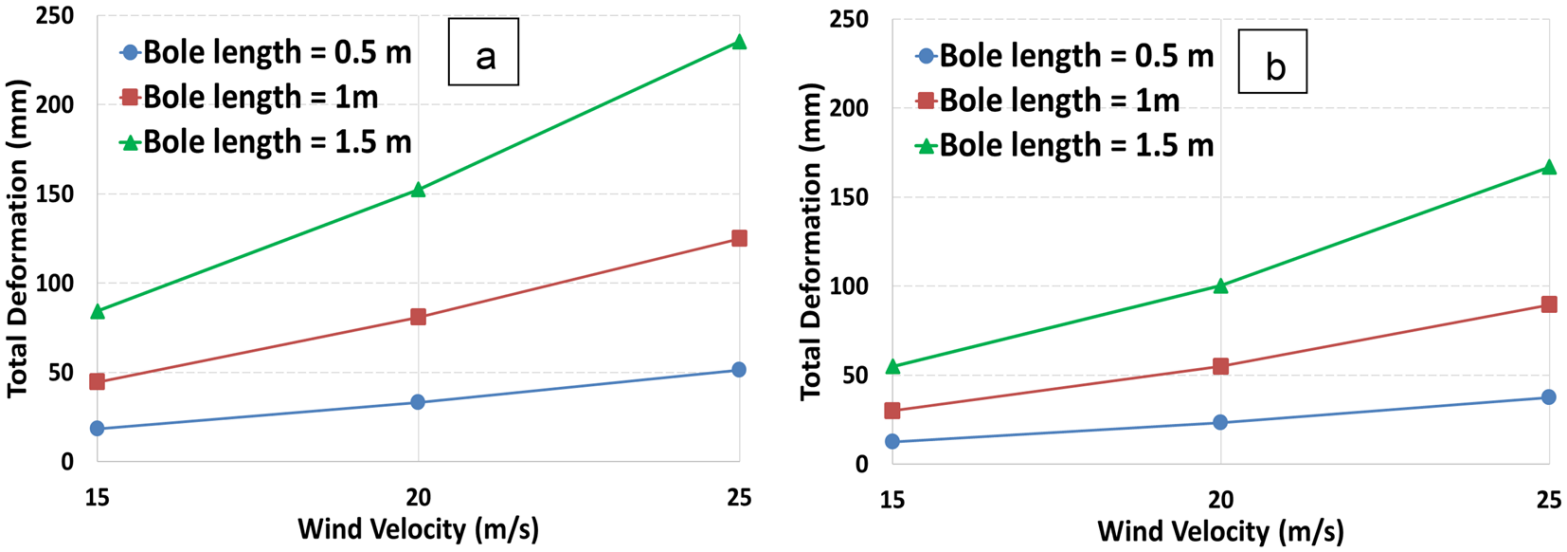
Total deformation varies in relation to various wind velocities for two tree models, model 1 (**a**) and model 2 (**b**).

In Fig. 5, the total deformations of the models 1 and 2 with different bole lengths are compared. It can be shown that bole length and canopy diameter have a significant effect on the total deformation. In the same trend, with an increase in wind velocity, this deformation difference with variable length increases considerably. The reduction trend is the same for both models, though the total deformation for model 2 for all bole lengths reduces in comparison with model 1. The deformation of tree models 1 and 2 increased with increasing wind velocity and bole canopy. Maximum deformation occurs when wind velocity and bole length are at their maximum values for models 1 and 2.

Van Mises stress can be computed in a three-dimensional flexible solid structure numerically. Van Mises stress is obtained using Eq. (9).

9 $${\upsigma }_{\mathrm{e}}={\left[\frac{{\left({\upsigma }_{1}-{\upsigma }_{2}\right)}^{2}+{\left({\upsigma }_{2}-{\upsigma }_{3}\right)}^{2}+{\left({\upsigma }_{3}-{\upsigma }_{1}\right)}^{2}}{2}\right]}^{1/2},$$

where ${\upsigma }_{1}$, ${\upsigma }_{2}$ and ${\upsigma }_{3}$ are stress states in the x, y, and z directions, respectively.

**Figure 6:**
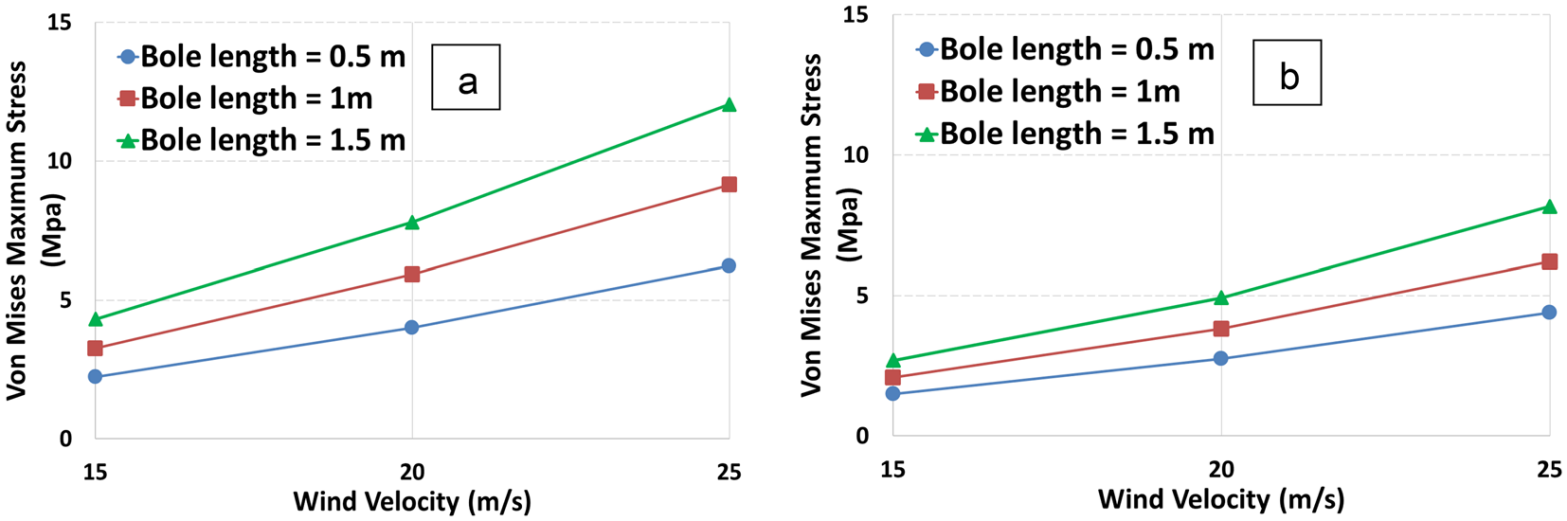
Von Mises maximum stress varies in relation to various wind velocities for two tree models, model 1 (**a**) and model 2 (**b**).

Figure 6 depicts the values of Von Mises maximum stress for different wind velocities and bole lengths. It is obvious that the wind velocity has a significant effect on maximum stress in models 1 and 2. As the velocity increases to 25 m/s, the maximum stress increases as much as three times. Moreover, it can be concluded that the bole length effect is more obvious in model 1 than model 2.

Equivalent strain can be computed numerically in a three-dimensional flexible solid structure. Equivalent strain is obtained using Eq. (10).

10 $${\upvarepsilon }_{\mathrm{e}}=\frac{1}{1+{\upupsilon }^{\mathrm{^{\prime}}}}{\left(\frac{1}{2}\left[{\left({\upvarepsilon }_{1}-{\upvarepsilon }_{2}\right)}^{2}+{\left({\upvarepsilon }_{2}-{\upvarepsilon }_{3}\right)}^{2}+{\left({\upvarepsilon }_{3}-{\upvarepsilon }_{1}\right)}^{2}\right]\right)}^\frac{1}{2},$$

where ${\upvarepsilon }_{1}$, ${\upvarepsilon }_{2}$ and ${\upvarepsilon }_{3}$ are principal strains in the in the x, y, and z directions, respectively.${\upsilon }^{\mathrm{^{\prime}}}$ represents effective Poisson’s ratio.

**Figure 7:**
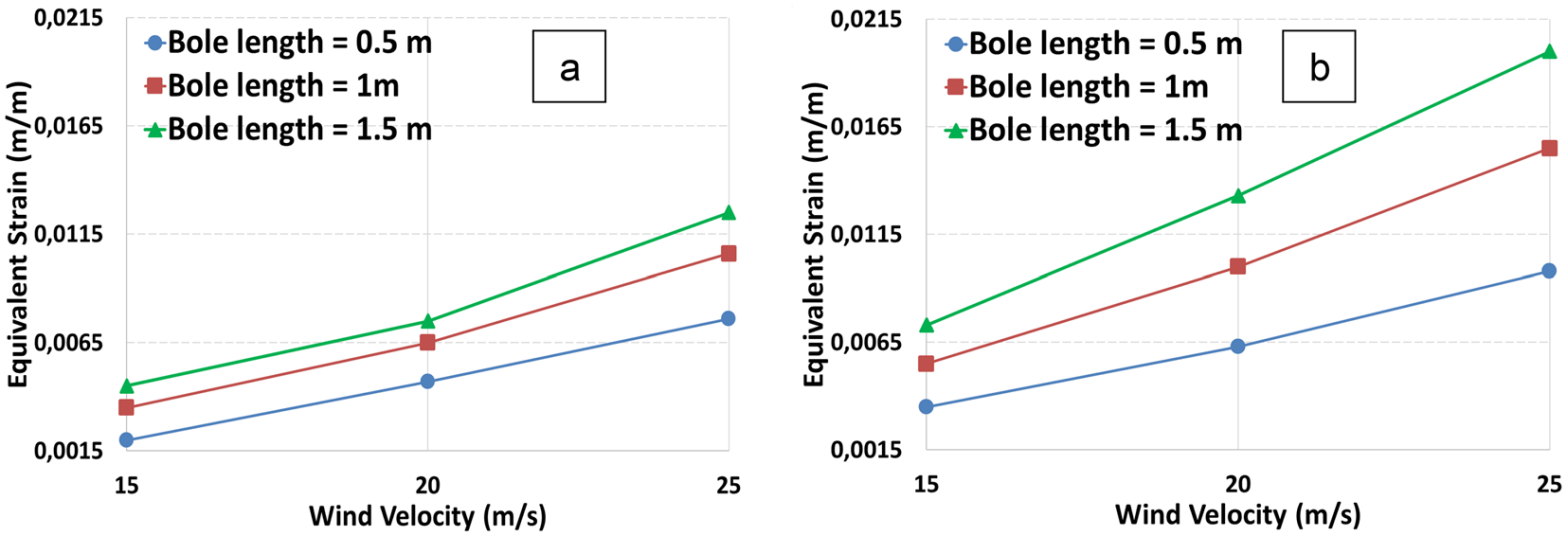
Equivalent strain varies in relation to various wind velocities for two tree models, model 1 (**a**) and model 2 (**b**).

Figure 7 depicts the equivalent strain with respect to wind velocity for different bole lengths. The strain is directly proportional to the bole length and wind velocity for both models. As the bole length increases, the strain increases as well. The increase is also dependent on the diameter.

### Contours plots of numerical study

**Figure 8:**
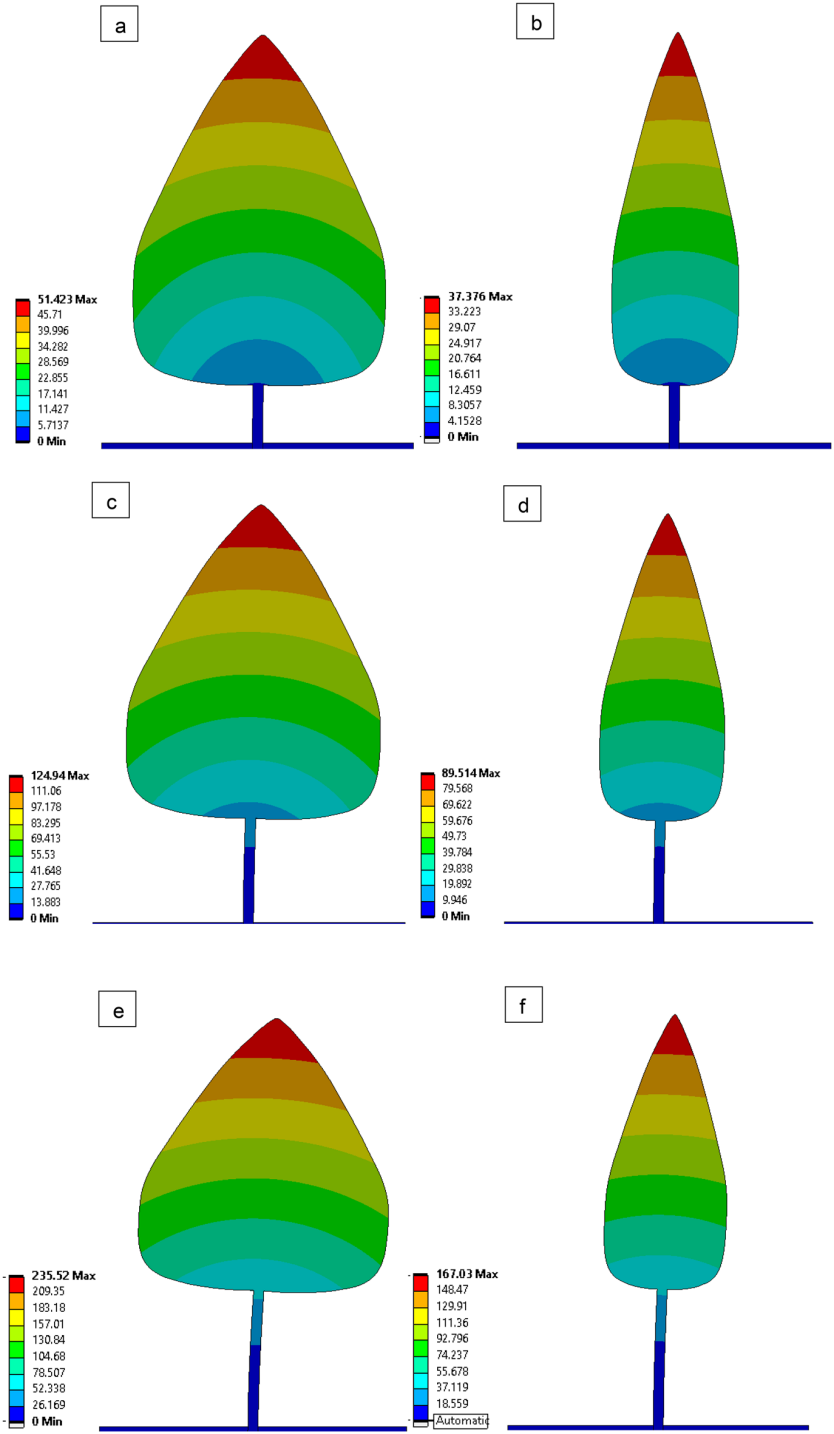
Deformation (mm) plots of tree models 1 and 2 at wind velocity 25 m/s, Bole Length = 0.5 m (**a,b**), Bole Length = 1.0 m (**c,d**), and Bole Length = 1.5 m (**e,f**).

**Figure 9:**
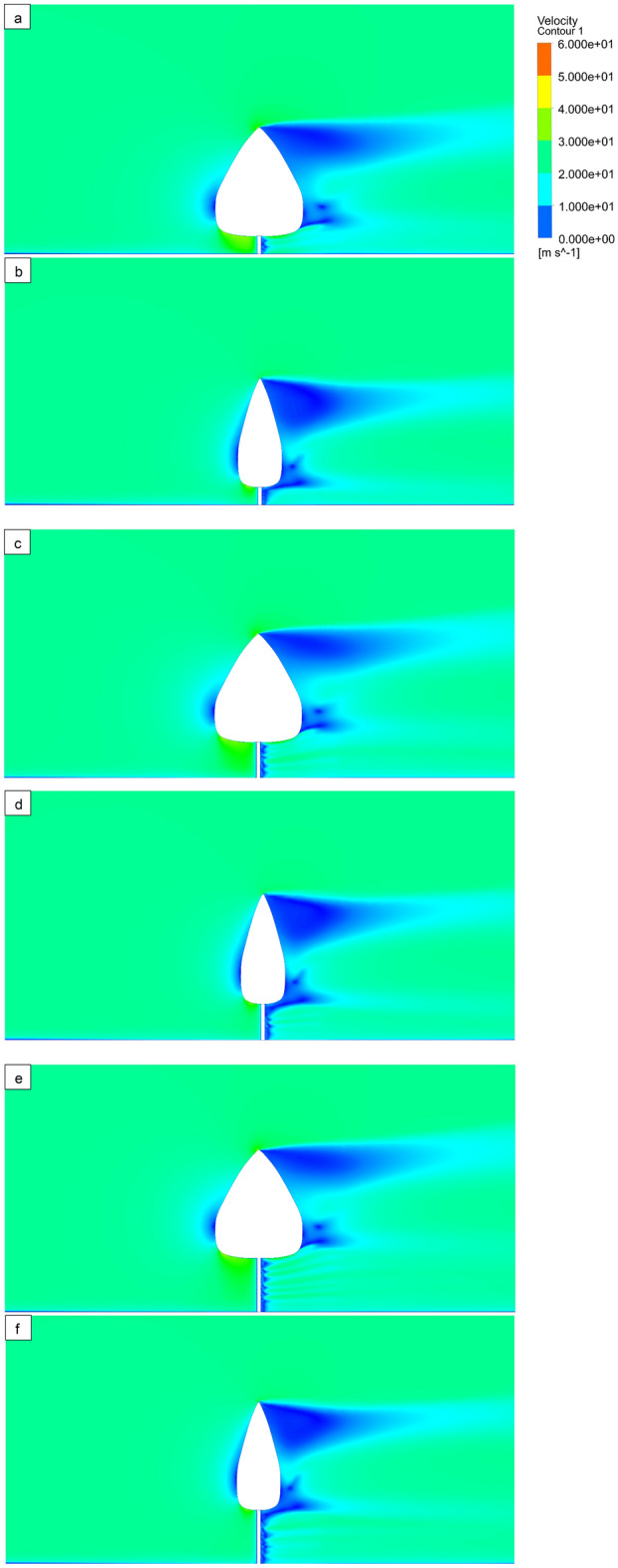
Velocity distribution (m/s) contour plots of tree models 1 and 2 at 25 m/s wind velocity, Bole Length = 0.5 m (**a,b**), Bole Length = 1.0 m (**c,d**), and Bole Length = 1.5 m (**e,f**) Bole Length = 0.5 m (**a,b**), Bole Length = 1.0 m (**c,d**), and Bole (**e,f**).

**Figure 10:**
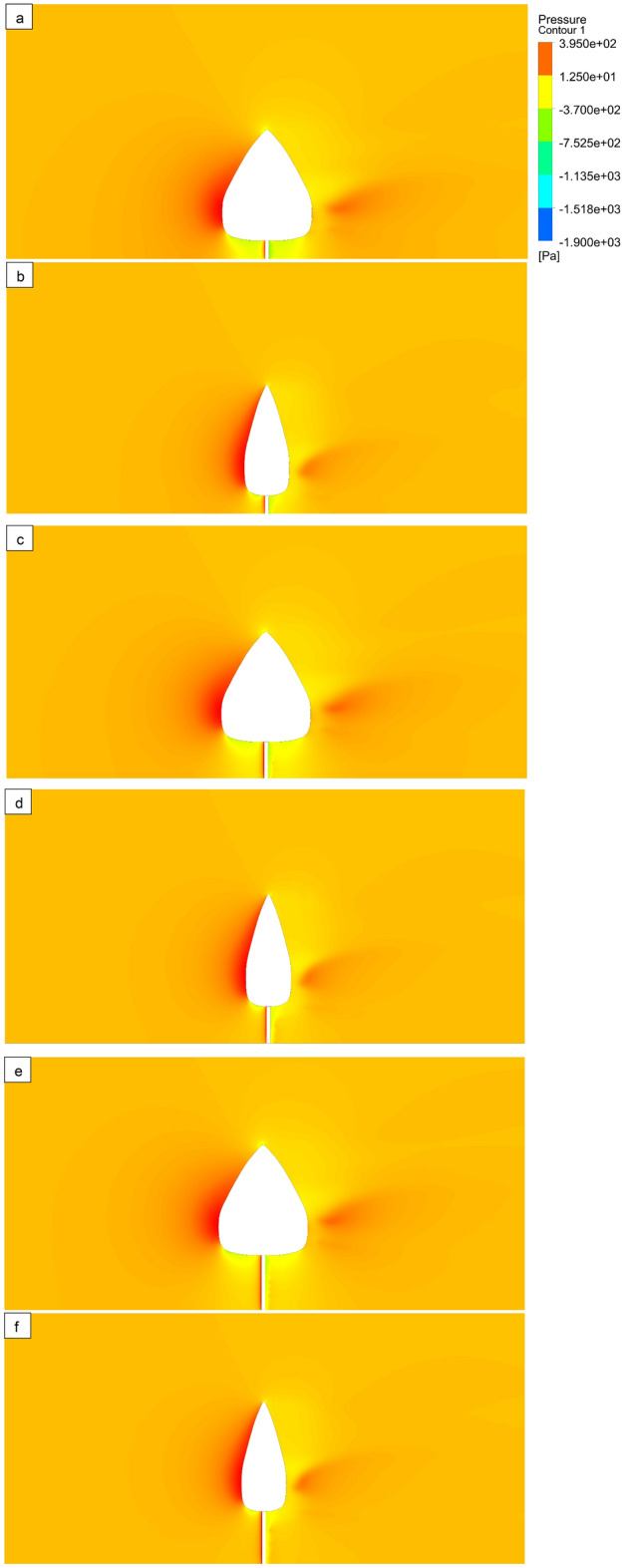
Pressure distribution (Pa) contour plots of tree models 1 and 2 at wind velocity 25 m/s, Bole Length = 0.5 m (**a,b**), Bole Length = 1.0 m (**c,d**), and Bole Length = 1.5 m (**e,f**).

The deformation of tree models is illustrated for various bole lengths (0.5 m, 1 m, and 1.5 m) and various wind velocities (15 m/s, 20 m/s, and 25 m/s) (Fig. 8). The velocity contours of the computational domain are plotted in the (Fig. 9) for different bole lengths (0.5 m, 1 m, and 1.5 m) and different wind velocities (15 m/s, 20 m/s, and 25 m/s). The minimum velocity occurs on the front surface of all tree models because high pressure is created in the front of the tree. Pressure contours of the computational domain are drawn in Fig. 10 for various bole lengths (0.5 m, 1 m, and 1.5 m) and various wind velocities (15 m/s, 20 m/s, and 25 m/s). It was noted that the large pressure happens on the front surface of the trees. It was noted that the large pressure regions happen on the front surface of the tree for all models. The lower pressure regions occur at the top of the tree and between the bottom of the tree and the ground. It was also realized that the pressure on model 1 is greater than on model 2 because of the larger surface.

## Conclusions

In this study, two different three-dimensional tree models were examined numerically using various body dimensions. Models 1 and 2 were evaluated under different wind velocities (15 m/s, 20 m/s, and 25 m/s) while varying the bole length from 0.5 to 1.5 m. The realizable k-e turbulence model was used to solve the fluid domain, and a one-way FSI method was employed to calculate both the fluid and solid domains together. The results of the total force, deformation, maximum stress, maximum strain, velocity distribution, and pressure distribution of both tree models were obtained at various geometry parameters and wind velocities. The results indicate that total force, deformation, stress, and strain increased with increasing wind velocity for both models. Additionally, deformation and stress were directly influenced by bole length, canopy diameter, and wind velocity when trunk diameter was constant. It was also observed that a high total force can cause significant deformation and stress in a tree. Model 1 demonstrated a greater total force than model 2 due to its larger diameter. The greatest deformation occurred at a wind velocity of 25 m/s and a bole length of 1.5 m for both models 1 and 2. Overall, it can be concluded that the chosen model tree can effectively hinder wind force, but it is important to note that parameters associated with the physical properties of the tree, such as length and diameter, can affect this function. The results of this study can be applied to urban planning and design by providing valuable information on how trees react to wind loading and which factors determine their effectiveness as windbreaks. This knowledge can help to improve the selection and placement of trees in urban areas, making the environment more comfortable for residents by reducing wind velocities.

## Data Availability

The datasets used and/or analyzed during the current study available from the corresponding author on reasonable request.
